# Inclusion of *Azolla pinnata* as an unconventional feed of Zaraibi dairy goats, and effects on milk production and offspring performance

**DOI:** 10.3389/fvets.2023.1101424

**Published:** 2023-02-20

**Authors:** Hanan A. M. Hassanein, Aristide Maggiolino, Magdy H. Abou El-Fadel, Pasquale De Palo, Heba A. El-Sanafawy, Ahmed M. Hussein, Abdelfattah Z. M. Salem

**Affiliations:** ^1^Animal Production Research Institute, Agricultural Research Center, Giza, Egypt; ^2^Department of Veterinary Medicine, University of Bari A. Moro, Valenzano, Italy; ^3^Facultad de Medicina Veterinaria y Zootecnia, Universidad Autónoma del Estado de México, Toluca, Mexico

**Keywords:** sun-dried azolla meal, lactating goats, digestibility, milk performance, milk composition

## Abstract

**Introduction:**

This study investigated the effects of using sun-dried Azolla (*Azolla pinnata*) meal (SDAM) protein to replace sunflower meal protein in the diets of Zaraibi goats dams on nutrient digestibility, milk yield, composition, and economics.

**Method:**

A total of 15 Zaraibi goats (32.23 ± 0.2 kg) were randomly divided into three equal groups, R1, R2, and R3 which were fed based on average milk production. The basal ration was a concentrated feed mixture containing 0, 10, and 20% SDAM which replaced 0, 25, and 50% of sunflower meal protein in the respective groups.

**Results:**

Nutrient digestibility and feeding values were improved with R3 goats, which had the highest level of azolla (20%) R3 versus R2 and R1 goats. The total volatile fatty acid (TVFA) concentration in the in-rumen liquor was elevated by increasing the level of azolla up to 20% in R3 goats. The results revealed significantly higher (*P*<0.05) mean milk yield in the SDAM groups in comparison to R1 (1184, 1131 and 1034 respectively). The beneficial effects of the tested groups were observed in milk composition, milk fat, milk protein, and non- fats solids. Whereas the milk fat yield was higher in the SDAM group in comparison with the control group (40.84, 37.20, and 33.92). Ration inclusion of SDAM improved economic feed efficiency (relative feed cost and relative daily profit) and had a significant effect on the yield of milk constituents. In general, using up to a level of 20% SDAM in place of sunflower meal for feeding lactating Zaraibi goats improved milk production, milk fat yield, and cost-benefit ratio.

**Discussion:**

This study recommended that, inclusion of sun-dried azolla meal up to 20%, as an unconventional feed for Zaraibi dairy goats and offspring, improved milk production and economically feed efficiency.

## Introduction

In many developing countries goats are important livestock used for meat and milk production. Compared with cow milk, goat milk from goats has a longer shelf life and is more easily digested. People who have complaints which prevent them from cow milk may reduce these issues through goat milk consumption (1, 2). As with other dairy products, animal nutritional changes can be reflected in the composition of goat milk and production economics (3). In Egypt, there is a lack of sufficient feed, particularly protein sources. To bridge this gap, unconventional feed resources must be used without compromising the quality of the nutrient supply. Azolla (*Azolla pinnata*) is a small floating aquatic fern with a symbiotic relationship with the cyanobacteria *Anabaena* Azolla, which can fix atmospheric N_2_ (4). Azolla has attracted the attention of scientists as the best alternative feed resource for livestock. The proximate composition of sun-dried azolla meal revealed that it is rich in crude protein, essential amino acids, ß-carotene, vitamins A and B12, minerals, and growth promoter intermediates. Moreover, it is easily digested by livestock due to its low lignin content and its high fiber content (5, 6). Therefore, azolla could be used as an unconventional protein source in livestock feed (7) and can be a potential feed ingredient for growing lambs. Additionally, it can partially replace concentrates used in livestock feed both fresh and dried and can be given mixed with concentrates or directly to goats without any adverse effects (8). Azolla improves the production of milk and meat in dairy cattle and is one of the most economical and efficient livestock feed substitutes (9). It also includes several valuable phytochemicals, amino acids, and fatty acids. These bioactive components contribute to a broad variety of useful and therapeutic properties, such as being antioxidant, anti-inflammatory anti-diabetic, and gastro-protective (10). Azolla meal can be included in the diet of growing lambs at a 10% content level replacing 25% from sunflower meal protein without any effect on the performance of the animals (11). The present study aimed to evaluate the effect of replacing sunflower meal protein with different inclusion levels of sun-dried azolla meal in concentrated feed mixtures on nutrient digestibility, milk yield, composition, and economic feed efficiency in lactating Zaraibi goats.

## Materials and methods

The present study was carried out at the Sakha Experimental Research Station of the Animal Production Research Institute (APRI), Agricultural Research Center, Kafer El-Sheik Governorate, Ministry of Agriculture, Egypt.

### Collection and preparation of azolla

Azolla was produced in 12.5 × 1.0 × 0.40 m. water troughs. Azolla was harvested Within 15 days. The period was from August to October in the year 2020. It was complete sun dried immediately after harvesting, then ground and mixed homogeneously with other feed ingredients to form concentrated feed mixtures.

### Experimental animals and feeding

The feeding trial lasted for 120 days (from 30 days at prepartum to 90 days postpartum). A total of 15 Zaraibi dairy goats (normal mating) were involved, with live body weights of 32.23 ± 0.2 kg and at third and fourth parity. Goats were randomly divided into three homogenous equal groups (five females each) according to their body weight, parity and milk production during the previous lactation season using a randomized complete block design. Goats were fed using a grouping system with berseem, fresh forage offered *ad libitum* and a basal ration where 8 kg/group from concentrate feed mixture (CFM) as described in NRC requirements (12). Animals tested the following feed rations: the control group (R1) received CFM1 containing 0% sun-dried azolla meal, while R2 and R3 received CFM2 and CFM3.containing 10 and 20% sun-dried azolla meal, respectively. All the rations were isonitrogenous and isocaloric. Animals were provided with rations divided into two feedings, morning and evening. Freshwater was freely available, and minerals and vitamin sources were offered in the cages to be licked by animals as needed throughout the experimental period. The ingredient composition of different CFMs is presented in Table 1. The nutritive values of CFM were 65% total digestible nutrients (TDN) and 14% crude protein (CP) approximately.

**Table 1:** Ingredient composition (%) of different concentrate feed mixtures (CFM).

**Ingredients**	**CFM1**	**CFM2**	**CFM3**
SDAM	–	10	20
Yellow corn grains	40	31	29
Soybean meal	–	–	2
Undecorticated sunflower meal (SFM)	20	15	10
Wheat bran	31	35	30
Molasses	5	5	5
Calcium carbonate	3	3	3
Salt	1	1	1

SDAM, sun dried azolla meal; CFM1, control; CFM2, 10% SDAM; CFM3, 20% SDAM.

#### Digestibility trial and rumen liquor parameters

Three digestibility trials (three doses per group) were conducted simultaneously with the animals involved during the last week of the feeding trial. The digestibility coefficients and feeding values of the tested ratios were determined according to the acid insoluble Ash (AIA) method as described previously (13). Faces sampled from the rectum twice daily within 12 h intervals for 5 consecutive days (10 samples per animal/group) were maintained at −20°C until analyzed. All samples of CFM, azolla, feces, and berseem were dried at 60°C for 72 h and analyzed according to (14) for dry matter (DM), crude fiber (CF), crude protein (CP), ether extract (EE), and ash content. Rumen liquor samples were collected using a stomach tube at zero time (before feeding) and 3 and 6 h post-feeding from three animals that fed on the experimental diets. pH level was immediately determined after rumen liquor was collected using a digital (Orian 680). Rumen liquor samples were filtered through four layers of cheesecloth. Ammoniacal nitrogen (NH3-N) concentration was measured according to (15). Total volatile fatty acid (TVFA) concentrations were measured according to (16). Calculated feed conversion included the amount of DM, total digestible nutrients (TDN) %TDN = %DCP + %DCF + %DNFE + (2.25^*^%DEE), digestible crude protein (DCP) units/kg of milk (DCP = Digestion coefficient CP^*^ CP). The economical evaluation was calculated for ratios according to the prevailing prices of ingredients and milk during 2020 the time of the experimental period. Price of kg raw milk: 5 LE/kg, CFM1:4035, CFM2:3615, CFM3:3310, and berseem (dry) 2,500 L.E./ton.

### Milk yield and feed utilization

Milk yield was recorded every 15 days. Moreover, the total milk yield was calculated by differences between weight of kids before and after suckling. Summation of milk yield along suckling period. Fifteen milk samples were collected at the middle of suckling period (at 45th day) were analyzed for fat, protein (%), and total solids parameters according to (17). Lactose (%) was determined by the calorimetric method according to (18). Ash content was estimated as described by (14). Solids-not-fat (SNF) values were calculated. The value of fat- corrected milk 4% (FCM) was calculated as FCM = 0.4 × milk yield (kg) + 15 × fat yield (kg) according to (19).

### Blood parameters

Blood samples were aseptically obtained *via* jugular vein puncture at the end of the collection period of the digestibility trial, Disposable needles (23G) with a negative pressure system were used for plasma (9 mL tubes with 15 USP U/mL of heparin) (Vacutainer^®^, Becton, Dickinson Canada Inc., Oakville, Canada) as described by (20). The blood samples were quickly centrifuged at 3,000 rpm for 20 min. The plasma fraction was frozen and stored at −20°C until analyzed. Different chemical parameters were assayed using commercial diagnostic kits. Plasma total proteins were determined according to (21). Albumin was determined according to (22), and globulin was calculated from the differences. Urea was estimated according to (23) while AST and ALT were also determined (24, 25).

### Statistical analysis

All data records were subjected to statistical analysis using SAS (26), with the general linear model denoted as:


$$\begin{array}{l} Y_{\text{ij}}=\mu +T_{\text{i}}+e_{\text{ij}} \end{array}$$

Where Y_ij_ is the observed value of the dependent variable determined from a sample taken from each animal; μ is the overall mean, T_i_ is the treatment effect (i = 1–3), and e_ij_ is the residual random error. Percentage data were subjected to arc-sin transformation to approximate the normal distribution before using the analyzed data. Significance was set as at *P* < 0.05 according to (27).

## Results

### Chemical composition

Results revealed that the chemical composition of SDAM contained 85.08, 18.58, 32.17, 3.35, 18.75, and 27.14% for DM, CP, CF, EE, NFE, and ash, respectively. The R3 group had the lowest values of (OM and NFE), although it had the highest values of DM, CP, CF, EE, NFE, and ash percentage, which were increased by increasing replacing undecorated sunflower meal with increasing levels of SDAM in the CFM groups (Table 2).

**Table 2:** Chemical composition^a^ of feed ingredients of tested diets (% per DM basis).

**Item**	**DM**	**OM**	**CP**	**CF**	**EE**	**NFE**	**Ash**
**Feedstuffs**							
SDAM^b^	85.08	72.86	18.58	32.17	3.35	18.76	27.14
SFM	86.94	95.54	37.68	19.89	1.52	36.45	4.46
Berseem	15.45	88.41	16.31	25.03	1.36	45.71	11.59
**Concentrate feed mixtures (CFM)**							
CFM1	88.35	92.89	15.56	7.95	2.89	66.49	7.11
CFM2	85.01	92.26	15.77	11.72	2.86	61.91	7.74
CFM3	88.05	83.58	15.64	14.21	3.11	50.63	16.42
**Experimental rations** ^c^							
R1	30.68	90.49	13.62	17.45	2.6	56.88	9.51
R2	30.62	90.08	14.11	19.59	2.6	53.78	9.92
R3	31.75	89.9	14.75	20.69	2.76	51.7	10.1

^a^DM, dry matter; OM, organic matter; CP, crude protein; CF, crude fiber; EE, ether extract; NFE, nitrogen free extract.

^b^SDAM, sun-dried azolla meal; CFM1, control; CFM2, 10% SDAM; CFM3, 20% SDAM; SFM, sunflower meal.

^c^Calculated, R1, Berseem + CFM1; R2, Berseem + CFM2; R3, Berseem + CFM3.

Nutrient digestibility coefficients and feeding values of tested feedstuff and rations.

The SDAM integrated with evaluated rations showed increasing digestibility coefficients for OM, DM, CP, and NFE. The nutritive values of TDN and DCP with R2 and R3 rations recorded the highest values (64.66 and 63.06% TDN, respectively) vs. 11.14 and 10.16% DCP for the respective rations, while R1 recorded 60.61% TDN and 9.53% DCP. The R1 differed (*P* < 0.05) from the other tested groups in digestion coefficients of DM, CF, EE, CP, and DCP. In contrast, the digestion coefficients of OM, NFE, and TDN showed no significant differences between the R1, R2, andR3 groups. The digestion coefficients of CF and EE for R2 were highest than those for the other groups. The R2 differed (*P* < 0.05) with R1 in the digestion coefficients of DM, EE, and DCP (Table 3).

**Table 3:** Effects of including azolla on digestibility coefficients and feeding values of experimental rations.

	**R1**	**R2**	**R3**
**Digestibility coefficient, %**			
Dry matter	61.73^b^ ± 1.46	67.57^a^ ± 1.46	64.96^ab^ ± 1.46
Organic matter	64.41 ± 1.38	69.01 ± 1.38	67.31 ± 1.38
Crude protein	69.96^b^ ± 1.97	78.94^a^ ± 1.97	68.87^b^ ± 1.97
Crude fiber	54.31^b^ ± 1.87	66.34^a^ ± 1.87	61.40^a^ ± 1.87
Ether extract	67.83^b^ ± 1.28	76.76^a^ ± 1.28	72.74^a^ ± 1.28
Nitrogen free extract	66.16 ± 1.54	67.01 ± 1.54	69.02 ± 1.54
**Feeding values, %**			
TDN	60.61 ± 1.26	64.66 ± 1.26	63.06 ± 1.26
DCP	9.53^b^ ± 0.28	11.14^a^ ± 0.28	10.16^b^ ± 0.28

Mean in the same row with different superscripts (a, b) are significantly different (*P* < 0.05).

TDN, total digestible nutrients; DCP, digestible crude protein.

DMD%= [1–AIA% of feed DM/AIA% of feces DM]^*^100.

Y = 100–{N/M(100-DMD)}. Where: DMD, dry matter digestibility; Y, nutrient digestibility; M, nutrient in feed, %DM; N, nutrient in feces, %DM.

### Rumen fermentation

Goats fed R2 and R3had values in the normal ranges for healthy animals. At various sampling times, all measured ruminal parameters were in the normal ranges. All values of ruminal pH always showed no significant differences among the experimental groups and the lowest values were seen at 3 h. The TVFA values increased until 3 h and declined at 6 h according to the normal distribution curve. The highest values of ruminal TVFA concentration (*P* < 0.05) was observed with the R2 and R3 groups compared with R1. The inclusion of SDAM led to an increase in NH3-N values at 3 h post-feeding. Although the values of NH_3_-N were non- significant among the tested groups at sampling times, the increasing SDAM levels increased the numerical values of NH_3_-N concentrations (Table 4).

**Table 4:** The effect of the inclusion of azolla on feed in rumen liquor parameters of Zaraibi dairy goats.

	**R1**	**R2**	**R3**
**pH values**			
0 h	6.69 ± 0.07	6.56 ± 0.07	6.60 ± 0.07
3 h	5.53 ± 0.14	5.47 ± 0.14	5.42 ± 0.14
6 h	6.19 ± 0.14	6.38 ± 0.14	6.65 ± 0.14
**NH**_3_ **-N, mg/dL**			
0 h	12.25 ± 1.42	14.63 ± 1.42	16.27 ± 1.42
3 h	31.15 ± 3.15	35.23 ± 3.15	36.21 ± 3.15
6 h	17.87 ± 3.29	21.93 ± 3.29	25.20 ± 3.29
**TVFA, meq/dL**			
0 h	09.01^b^ ± 0.41	11.43^a^ ± 0.41	10.30^ab^ ± 0.41
3 h	15.54 ± 1.77	17.76 ± 1.77	17.71 ± 1.77
6 h	13.58 ± 0.88	15.75 ± 0.88	13.48 ± 0.88

Mean in the same row with different superscripts (a, b) are significantly different (*P* < 0.05).

### Blood parameters

The goats fed R3 diet had higher (*P* < 0.05) levels of plasma total protein, albumin, and ALT compared with R1 and R2. Goats of R3 had the highest value of creatinine (1.61 mg/dl *P* < 0.05) which differed (*P* < 0.05) from other groups. A significant effect with the lower concentrations was recorded in R1 and R2 groups (1.30 and 1.39 mg/dl, respectively) compared to the R3 group. The AST and ALT levels in plasma indicated that goats fed the test diets had sufficient nutrients for their maintenance and sustained milk production (Table 5).

**Table 5:** Blood biochemical constituents affected by feeding sun-dried azolla meal in rations of Zaraibi dairy goats.

**Parameters**	**R1**	**R2**	**R3**
Total protein, g/dl	06.76^b^ ± 0.12	06.89^b^ ± 0.12	07.40^a^ ± 0.12
Albumin, g/dl	03.84^b^ ± 0.07	03.85^b^ ± 0.07	04.23^a^ ± 0.07
Globulin, g/dl	03.00 ± 0.13	03.04 ± 0.13	03.17 ± 0.13
ALT, IU/l	14.82^b^ ± 0.33	15.72^b^ ± 0.33	16.89^a^ ± 0.33
AST, IU/l	25.49 ± 0.47	28.53^a^ ± 0.47	29.80^a^ ± 0.47
Urea, mg/dl	07.29 ± 0.10	07.46 ± 0.10	07.27 ± 0.10
Creatinine, g/dl	01.30^b^ ± 0.05	01.39^b^ ± 0.05	01.61^a^ ± 0.05

Means in the same row with different superscripts (a, b) are significantly different (*P* < 0.05).

### Milk yield, milk chemical composition

Actual milk and 4% FCM yields were higher (*P* < 0.05) in R3 vs. R1 goats. Meanwhile, the highest value for actual milk yield was found in R3 (1,184 g/h/d), with no difference between R2 and R3 goats. No significant effects on milk composition were seen among goat's whose diet included SDAM. Additionally, milk constituent yields had no significant effect from the tested rations. Fat percentages in milk had an insignificant effect with 3.28, 3.29, and (3.45 ± 0.20) for R1, R2, and R3 respectively. There was no significant difference between the R1, R2, and R3 goats shown in the average percentage of milk composition (Table 6).

**Table 6:** Effects of the inclusion of azolla (SDAM) unconventional feed on milk yields and milk composition of Zaraibi dairy goats.

	**R1**	**R2**	**R3**
Actual daily milk yield, g/ h/d	1034^b^ ± 26.68	1131^a^ ± 26.68	1184^a^ ± 26.68
4%FCM yield, g/ h/d	0922^b^ ± 39.09	1017^ab^ ± 39.09	1090^a^ ± 39.09
**Milk composition, %**			
Fat, %	03.28 ± 00.20	03.29 ± 00.20	03.45 ± 00.20
Protein, %	02.55 ± 00.26	02.38 ± 00.26	03.54 ± 00.26
Lactose, %	03.81 ± 00.15	03.88 ± 00.15	03.88 ± 00.15
Total solids, %	10.59 ± 00.48	10.47 ± 00.48	11.87 ± 00.48
SNF, %	07.30 ± 00.33	07.18 ± 00.33	08.42 ± 00.33
Ash	00.85 ± 00.04	00.92 ± 00.04	01.00 ± 00.04
**Milk constituent yield, g/h/d**			
Fat	33.92 ± 02.37	37.20 ± 02.37	40.84 ± 2.37
Protein	26.37 ± 04.26	26.92 ± 04.26	41.91 ± 4.26
Lactose	39.40 ± 02.64	43.88 ± 02.64	45.94 ± 2.64
Total solid yield	109.50 ± 08.11	118.42 ± 08.11	140.54 ± 8.11
Solids not fat yield	75.48 ± 06.77	81.21 ± 06.77	99.69 ± 6.77

Means in the same row with different superscripts (a, b) are significantly different (*P* < 0.05).

### Dams and their offspring performance

Data presented in Table 7, show the effect of experimental rations on dams and their offspring performance. There were insignificant differences between R1, R2, and R3 in most dams' parameters. Generally, neither before nor after lambing were significant changes in body weight seen in the dams among the tested groups. Gradual increases in dams body weight of 29.18, 28.2, and 30.4 kg after kidding, to 30.6, 30.2, and 30.6 kg at weaning for R1, R2, and R3, respectively, were observed. Kids in group R2 showed an average daily gain of 133.33 g, and the highest weaning weight of 14.50 kg. Data for offspring performance measurements, particularly for kid birth weight revealed a few differences among the treatments. Data concerning of litter weight at weaning per dam showed that no significant differences between experimental groups with higher values in R3.

**Table 7:** Performance effects of feeding dams and their offspring sun dried azolla meal rations.

	**R1**	**R2**	**R3**	**±SE**
**Dams' performance**				
Number of dams kidded	5	5	5	
Initial weight, Late-pregnancy kg	31.95	32.40	32.35	2.67
Body weight at parturition, kg	29.18	28.20	30.40	2.13
Body weight at 1st month after parturition, kg	23.20	24.80	25.80	1.48
Body weight at 2nd month after parturition, kg	26.90	27.40	27.80	1.56
Body weight at 3rd month after parturition, kg	30.60	30.20	30.60	1.45
**Offspring performance**				
Total number of kids	8	8	9	
Litter size /dam at birth (LSB)	1.60	1.60	1.80	0.24
Birth weight, kg	2.49	2.50	2.39	0.05
Weight after 45 days, kg	10.00^a^	7.40^b^	7.94^b^	0.28
Weaning weight, kg	13.63	14.50	14.11	0.51
Total weight gain, kg	11.14	12.00	11.72	
Average daily gain, g/day	123.78	133.33	130.22	5.46
Relative improvement (%)	100.00	107.72	105.20	
**Dam production**				
Litter weight at birth, kg	3.98	4.00	3.86	0.56
Litter weight at weaning, kg	21.80	23.20	25.40	3.75
Total litter weight gain, kg	17.82	19.20	21.54	
Average daily gain, g/day	198.0	213.33	239.33	
Relative improvement, %	100	108	120	

Mean in the same row with different superscripts (a, b) are significantly different (*P* < 0.05).

### Feed efficiency of milk production and economic efficiency

The inclusion of SDAM in diets did not affect DM intake, while TDN and DCP intake (g/h/d) were elevated vs. the R1 group due to increasing SDAM in the R2 and R3 groups. These results may be due to higher TDN and DCP contents in rations containing more SDAM than in R1 goats. Improved feed conversion was observed in goats fed SDAM as higher kg DM intake/kg milk and kg DCP intake/ kg milk when compared with those of R1. However, improvement in kg TDN intake/kg milk was observed only with R2, and this may be due to increased milk production. Goats of R3 showed the best improvement in the relative feed cost of rations containing SDAM. As a result, feeding ratios containing SDAM, daily profit, economic efficiency, and relative economic efficiency were all improved. Data economics of feed efficiency showed that the feeding cost per kg of milk was decreased with increased azolla levels in the diet. The lowest cost of feeding per kg of milk was observed in the R3 goats (Table 8).

**Table 8:** Effects of inclusion of azolla on daily feed intake, feed conversion, and economic efficiency.

**Item**	**R1**	**R2**	**R3**
**Average feed intake, g/h/d (as fed)**			
CFM	707	680	704
Berseem	575	563	523
Total DM intake, g/h/d	1,282	1,243	1,227
TDN intake, g/h/d	774	802	780
DCP intake	122	138	124
**Feed conversion**			
DM intake, kg/kg (4%FCM) milk	1.39	1.22	1.13
TDN intake, kg/kg (4%FCM)	0.839	0.789	0.716
DCP intake, g/kg (4%FCM)	132	136	113
**Economic evaluation** ^a^			
Price of average daily milk, LE/dam/day	5.17	5.66	5.92
**Average daily feed cost, LE/ dam/day**			
CFM	2.85	2.45	2.33
Berseem	1.44	1.41	1.31
Total feed cost, LE/dam/day	4.29	3.86	3.64
Relative feed cost^b^, %	100	89.97	84.85
Feed cost/kg milk, L.E.	4.14	3.41	3.07
Daily profit, LE/goat/day	0.88	1.80	2.28
Relative daily profit, %	100	205	259

^a^Prices of CFM1: 4,035 LE/ton; CFM2: 3,615 LE/ ton; CFM3: 3,310 LE/ ton; and berseem (dry) 2,500 L.E./ton. These price are according to the market in 2019 price of kg raw milk: 5 LE/kg.

^b^Relative cost% = the cost tested ration/the cost of control ration.

Relative cost % = (the cost tested ration/the cost of control ration) ^*^ 100, Feed cost /kg milk L.E. = feed cost/milk yield kg. Daily profit L.E/goat/day = (milk yield kg ^*^ price of 1 kg milk) – feed cost.

Relative daily profit % = (the daily profit LE/goat/day for tested ration/the daily profit of control ration) ^*^ 100.

## Discussion

The DM content and OM of SDAM showed the same trends as observed previously (28, 29), while the CF, ash, EE, and DM levels showed the same trend as seen by Bhatt et al. (30). The high protein content could be due to the high nitrogen content fixed by the bacterium azolla. The nutrient composition of azolla varies according to environmental conditions during cultivation (5). Furthermore, the ash percentages of SDAM, OM, and NFE were associated as reported by Shukla et al. (31), who also found that an increased azolla rations, decreased OM content but increased CF this may be due to the higher total ash and CF content of azolla. In contrast, the percentage of CF of SDAM observed disagree with observations by Ara et al. (32).

Significantly higher DM digestibility was seen in the azolla-based diet than in the control group. Similarly, the highest DM digestibility was observed previously in rations containing 6% azolla meal (33, 34). In contrast, it was reported that increasing the integration of azolla meal in rations of Osmanabadi kids decreased the digestibility of DM, CP, CF, EE, and NFE (35, 36). However, when 20% of CFM was replaced by azolla on an equivalent weight basis, NFE digestibility was lowered in Black Bengal goats (8). As reported, azolla enhances FCR, energy efficiency, and performance with no adverse effects on livestock (36), these results may be due to the azolla meal had higher content of curd fiber and ash.

High fermentation of carbohydrates has been shown to decreased pH values due to increases in TVFA production and higher digestibility of organic matter (37, 38) that was agree with our results for data of ruminal fermentation parameters.

Plasma total protein and its fractions are considered as a biological index reflecting productive performance and health of the animal. The present study results agree with previous findings (39, 40) and indicate normal ranges for the samples goats. Additionally, increases in the urea content in blood and milk have been reported to result from increasing intake of digestible crude protein or digestible crude protein/MJ (41). The value seen here was within the normal range and is also comparable with previous findings (42). In general, including up to 20% SDAM concentrate mixture in Zaraibi dairy goat feed resulted in no harmful effects to hematological and biochemical parameters.

Daily milk yield and its composition showed similar results as seen in lactating Barberi goats fed azolla, and their milk production increased 19.87% compared to goats fed a control diet (43). However, increased 10–15% in cattle and 19.32% in buffaloes fed fresh azolla (44, 45), which were 9.38 and 14.50% for 10 and 20% SDAM, respectively. The observed milk fat percentage results concur with several previous studies (45–49), and however, animals fed commercial feed combined with azolla increased both quantity (10–15%) and quality of milk (higher fat content) and showed improved animal health (46). Supplementing SDAM enhanced milk yield but had little effect on fat percentage and caused an increase in FCM yield. The differences in the chemical composition and production of milk may be due to the higher content of minerals and different bioactive substances in SDAM diets. These in turn may enhance digestibility and nourishment which then may stimulate milk production (5).

The higher post-partum weight indicates a higher birth weight for kids (47, 48), and furthermore, the growth rate is also affected by litter size. Bhatt et al. (49) found that the average daily live-weight gain among Sahiwal female calves was higher for groups with 15% followed by 30% feed content of *A. pinnata* on a DM basis. Similarly, it was noted that the growth rate was improved when replacing the concentrate with 5% *A. pinnata* (50). Additionally, the inclusion of sun-dried azolla up to 20%of the CFM of goat kids had no harmful effects on the performance, digestibility of nutrients, or carcass characteristics, and increased meat weight by 8–10% (51–54). Feed conversion efficiency was reduced with the inclusion of azolla meal (52), however, the present results were similar and correspond with observations by Sihag et al. (53) for DM intake, which showed higher ADG when the CFM was replaced with 10% azolla. Azolla, due to its high protein content, can play an important role in accelerating the growth of animals; thus, it can be used as a growth enhance.

DM intake per kid was observed to be greater with 15% azolla content (35), and lower FCR may be due to decreased DM intake (54). Feeding of 2 kg azolla instead of concentrate in crossbred calves was seen to reduce the milk production cost and feed labor costs by 18.5 and 16.6%, respectively (55), but in the present study, addition of SDAM at 10 and 20% in CFM reduce total feed cost/kg milk (LE) by 17.63 and 25.84%, respectively. A study of Osmanabadi kids concluded that the use of azolla meal is relatively beneficial when the total concentrate includes up to 15% azolla meal (35). Other studies have found that greater quantity of azolla used in feeding goat kids reduces feeding costs (56), and that the greatest output and the lowest feed costs occur when goats are fed a diet containing 15% fresh azolla (57). Additionally, a positive impact on the economic feed efficiency of growing crossbred lambs was found when up to 10% azolla meal was incorporated into diets in place of sunflower meal (11). However, the present study showed that the best percentage of SDAM at 20% in CFM was improved total economic feed efficiency parameters.

## Conclusion

Inclusion of sun-dried azolla meal up to 20% as an unconventional feed for Zaraibi dairy goats and offspring improvement milk production and economically feed efficiency.

## Data availability statement

The raw data supporting the conclusions of this article will be made available by the authors, without undue reservation.

## Ethics statement

The animal study was reviewed and approved by the Ethical Committee of the APRI, Agricultural Research Center, Egypt.

## Author contributions

HH, MA, HE-S, and AH designed the experiment and carried out the research and laboratory analysis. HH, MA, PD, AM, and AS conducted the data analysis, wrote the manuscript, and revised the manuscript. All authors reviewed and agreed on the final manuscript. All authors contributed to the article and approved the submitted version.
